# Association of fatty pancreas with pancreatic endocrine and exocrine function

**DOI:** 10.1371/journal.pone.0209448

**Published:** 2018-12-20

**Authors:** Hayato Miyake, Junichi Sakagami, Hiroaki Yasuda, Yoshio Sogame, Ryusuke Kato, Kanetoshi Suwa, Katsuyuki Dainaka, Tomoki Takata, Isao Yokota, Yoshito Itoh

**Affiliations:** 1 Department of Medicine, Division of Gastroenterology and Hepatology, Kyoto Prefectural University of Medicine, Kyoto, Japan; 2 Department of Biostatistics, Kyoto Prefectural University of Medicine, Kyoto, Japan; University of Szeged, HUNGARY

## Abstract

**Aim:**

The purpose of this study was to clarify whether fatty pancreas might lead to impaired pancreatic endocrine or exocrine function.

**Material and methods:**

The study involved 109 participants who had undergone the glucagon stimulation test and N-benzoyl-L-tyros-p-amino benzoic acid (BT-PABA) test to assess pancreatic function as well as unenhanced abdominal computed tomography (CT). Pancreatic endocrine impairment was defined as ΔC peptide immunoreactivity less than 2 [mmol/L] in the glucagon stimulation test, and pancreatic exocrine impairment was defined as a urinary PABA excretion rate less than 70% on the BT-PABA test. We defined as the mean CT value of pancreas / CT value of spleen (P/S ratio) as a marker to assess fatty pancreas. We analyzed the association between fatty pancreas and pancreatic impairment using the logistic regression model. The odds ratio (OR) is shown per 0.1 unit.

**Results:**

Pancreatic endocrine function was impaired in 33.0% of the participants, and 56.9% of those were regarded as having pancreatic exocrine impairment. The P/S ratio was significantly correlated with pancreatic endocrine impairment in univariate analysis (OR = 0.61, 95% confidence interval (CI) = 0.43–0.83, *P* = 0.0013) and multivariate analysis (OR = 0.38, 95% CI = 0.22–0.61, *P* < .0001) for all participants. Similar significant relationships were observed in both univariate (OR = 0.70, 95% CI = 0.49–0.99, *P* = 0.04) and multivariate (OR = 0.39, 95% CI = 0.21–0.66, *P* = 0.0002) analyses for the participants without diabetes (n = 93). The amount of pancreatic fat was not associated with exocrine impairment in univariate analysis (OR = 0.80, 95% CI = 0.59–1.06, *P* = 0.12).

**Conclusion:**

Fatty pancreas was associated with pancreatic endocrine impairment but did not have a clear relationship with pancreatic exocrine impairment.

## Introduction

In practical medicine, fatty pancreas is occasionally detected by ultrasonography, computed tomography (CT), or magnetic resonance imaging. Fatty pancreas is a general term for so-called pancreatic fat accumulation. However, some terms, such as “pancreatic lipomatosis” or “fatty infiltration”, specifically define processes relating to fat accumulation and the distribution of fat in the pancreas [1].

In the last few years, fatty pancreas has been recognized as a risk factor for several diseases. For example, the severity of acute pancreatitis is associated with pancreatic fat deposition [2]. Furthermore, the amount of pancreatic fat has been reported to be an independent risk factor for the development of pancreatic cancer [3].

However, it remains unclear whether fatty pancreas is related to impaired pancreatic endocrine and/or exocrine function.

First, the association between fatty pancreas and pancreatic endocrine impairment is controversial. One prior study in rats suggested that fat accumulation in pancreas may contribute to β-cell lipotoxicity with the consequent loss of β-cell function [4]. However, it is not clear whether this pathology occurs in humans. Wang et al. [5] found that individuals with fatty pancreas had an increased risk of diabetes (odds ratio (OR) = 1.593, 95% confidence interval (CI) = 1.300–1.953) compared to those with nonfatty pancreas (*P* < 0.001). Chai et al. [6] found a significantly higher average fat content of the pancreas in patients with type 2 diabetes than in healthy controls. In contrast, a study using a hyperglycemic clamp found no relation between fatty pancreas and β-cell function in participants with impaired glucose metabolism [7]. By using CT, Saisho et al. [8] observed that the levels of pancreatic fat were not significantly increased in patients with type 2 diabetes.

Second, the association between fatty pancreas and pancreatic exocrine impairment is also controversial. There are some case reports describing the association between pancreatic exocrine impairment and fatty pancreas [9,10]. Moreover, Tahtaci et al. also reported that the fatty pancreas assessed with magnetic resonance imaging might affect the pancreatic exocrine impairment [11]. However, the association was not shown in the studies targeting patients with diabetes [12,13].

The aim of our study was to clarify whether fatty pancreas may lead to impaired pancreatic endocrine and/or exocrine function. We selected patients who had undergone the glucagon stimulation test and/or the N-benzoyl-L-tyros-p-amino benzoic acid (BT-PABA) test to assess pancreatic endocrine and exocrine function, respectively.

In the glucagon stimulation test, an increase in the C-peptide immunoreactivity levels (ΔCPR) was measured after intravenous injection of glucagon. One reason why the association between fatty pancreas and pancreatic endocrine impairment is controversial is the difficulty of quantifying *in vivo* β-cell function in humans. Glucagon is a stimulus for β-cells, and an intravenous injection of 1 mg glucagon has been widely used to assess β-cell function. Fujita et al. [14] found that ΔCPR is closely correlated with the human relative β-cell area. However, the association between fatty pancreas and pancreatic endocrine impairment with the glucagon stimulation test has not been reported to date.

Chymotrypsin activity was assessed [15] *in vivo* with the BT-PABA test, which is the only method currently available in Japan to assess pancreatic exocrine function.

This study was expected to clarify the pancreatic endocrine and exocrine function of fatty pancreas, which has long been overlooked.

## Materials and methods

### Study population

This study was reviewed and approved by the Institutional Review Board of Kyoto Prefectural University of Medicine (the approval number: ERB-C-982). The Institutional Review Board of the Kyoto Prefectural University of Medicine approved a waiver of informed consent because this analysis used anonymous clinical data. The participants in this study were adults who had undergone the glucagon stimulation test and the BT-PABA test at the University Hospital, Kyoto Prefectural University of Medicine between 2008 and 2016 and who also had undergone nonenhancing CT of abdomen within 1 year of these tests. Individuals with neoplastic diseases, such as pancreatic cancer and autoimmune pancreatitis, were excluded because of the possibility that these diseases might influence the endocrine and exocrine function of the pancreas. Participants who had undergone pancreatectomy were also excluded for the same reason. However, those with suspected branch duct intraductal papillary mucinous neoplasms were included because these cystic regions were often difficult to distinguish from nontumorous cysts, such as retention cysts or simple cysts.

### Glucagon stimulation test and the definition of pancreatic endocrine impairment

Following the intravenous injection of 1 mg glucagon (Novo Nordisk, Tokyo, Japan), blood samples were obtained at 0 and 6 min for the determination of ΔCPR [mmol/L]. Pancreatic endocrine impairment was defined as ΔCPR less than 2 [mmol/L].

### BT-PABA test and the definition of pancreatic exocrine impairment

Orally administered BT-PABA is degraded with chymotrypsin, and the liberated PABA is absorbed in small intestine. After PABA undergoes the conjugation in liver, it is finally excreted in the urine. BT-PABA test is an examination to evaluate chymotrypsin activity by measuring urinary PABA excretion rate.

The first urine sample was collected as a control after an overnight fast. Then, the patients were orally given 500 mg BT-PABA (Eisai Co., Ltd, Tokyo, Japan) in 250 ml of water. The urinary PABA excretion rate [%] was calculated with the urine sample at 6 hours after BT-PABA administration. Pancreatic exocrine impairment was defined as a urinary PABA excretion rate less than 70%.

### Assessment of pancreatic and visceral fat

To assess the amount of pancreatic fat more easily and objectively, we first measured the CT values of pancreas on nonenhancing CT images. CT values of the pancreas were measured within the circular region of interest (ROI) comprising an area of 1 cm^2^ at the three different areas (head, body and tail of the pancreas). We set the ROIs as large as possible at the atrophic pancreas, which was a difficult location to establish 1-cm^2^ ROIs. When setting ROIs, calcifications, cysts, vascular structures, biliary ducts and pancreatic ducts were avoided. Then, we also measured the CT values of the spleen with a circular ROI as large as possible on the same CT images. To minimize the impact of bias in setting the ROI, we used a method to measure CT values of the spleen that was different from the one in previous reports, which establishes three ROIs in the spleen [16]. In order to validate the 1-ROI technique to assess the CT attenuation of spleen, two observers who were blinded to the clinical information of the participants assessed the CT attenuation of spleen with the 1-ROI technique, and intra-observer variation was assessed using intra-class correlation coefficients (ICC). Intra-observer for the 1-ROI technique was 0.96 (95% confidence interval:0.95–0.98). Therefore, we thought that the 1-ROI technique was the reliable method to assess the CT attenuation of spleen.

We finally defined the marker of pancreatic fat content as the mean CT value of the three ROI measurements in pancreas/CT value of spleen (P/S ratio) in each patient according to an existing assessment for hepatic fat content [17]^.^

We also measured the area [cm2] of visceral fat at the level of the umbilical region on the slice of the same CT series as that described in a previous report [18]

### Clinical data collection

The clinical data of the participants were extracted from electronic medical records. These data included age, sex, body mass index (BMI) [kg/m^2^], clinical diagnosis of pancreatic diseases, comorbidities and laboratory data (the results of the glucagon stimulation and BT-PABA tests). The comorbidities examined in this study were (1) diabetes with treatment history, (2) hypertension with treatment history, and (3) dyslipidemia with treatment history.

### Statistical analysis

Statistical analyses were performed with JMP version 11.2.0 (SAS Institute Inc., NC). The association between amount of pancreatic fat (P/S ratio) and pancreatic endocrine and/or exocrine impairment was assessed using univariate and multivariate logistic regression analysis. A *P* value less than 0.05 was considered statistically significant. The odds ratio (OR) is shown per 0.1 unit.

## Results

### Patient characteristics (Table 1)

A total of 109 cases were included in the analysis, as shown in Table 1; 66 (60.6%) of the patients were male, and the overall mean age was 65.8 years. Thirty-one patients had calcifications of the pancreas, including those who had a definitive diagnosis of chronic pancreatitis. In all cases, acute pancreatitis had been cured at the time of the tests. Pancreatic endocrine function was impaired in 33.0% of participants, and 56.9% of them were regarded as having pancreatic exocrine impairment.

**Table 1:** Characteristics of the participants.

	N	%	Mean ± SD [ranges]
Total	109		
Sex (male)	66	60.6	
Age			65.8 ± 12.7 [31 – 87]
Body mass index (kg/m^2^)			22.0 ± 3.5 [15.3–34.9]
Clinical diagnosis			
Cystic lesions of the pancreas	44	40.4	
Calcifications of the pancreas	27	24.8	
Cystic lesions and calcification of the pancreas	4	3.7	
After acute pancreatitis	6	5.5	
No obvious image findings of pancreatic diseases	28	25.7	
Comorbidities			
Diabetes	16	14.7	
Hypertension	29	26.6	
Dyslipidemia	20	18.3	
Laboratory data			
Glucagon stimulation test			
C-peptide immunoreactivity at 0 min (mmol/L)			1.6 ± 0.7 [0.03–4.49]
C-peptide immunoreactivity at 6 min (mmol/L)			4.4 ± 1.8 [0.08–9.80]
ΔC-peptide immunoreactivity (mmol/L)			2.8 ± 1.5 [0.05–7.91]
Pancreatic endocrine impairment	36	33.0	
BT-PABA test			
Urinary PABA excretion rate (%)			64.6 ± 14.6 [3.2–102.5]
Pancreatic exocrine impairment	62	56.9	
CT findings			
CT value of pancreatic head (HU)			45.8 ± 11.3 [9.0–92.9]
CT value of pancreatic body (HU)			45.0 ± 10.8 [14.4–92.9]
CT value of pancreatic tail (HU)			43.3 ± 9.9 [15.6–86.7]
Mean CT value of the pancreas (HU)			44.8 ± 9.6 [14.4–90.8]
CT value of spleen (HU)			51.3 ± 7.0 [42.0–97.1]
P/S ratio			0.87 ± 0.14 [0.34–1.18]
Visceral fat area (cm^2^)			71.1 ± 48.9 [5.5–217.6]

BT-PABA: N-benzoyl-L-tyros-p-amino benzoic acid, HU: Hounsfield unit, P/S ratio: mean CT value of pancreas/CT value of spleen, SD: standard deviation.

P/S ratio had mild association with visceral fat area (correlation coefficient; -0.36, *P* = 0.0001).

### Association between pancreatic endocrine impairment and the amount of pancreatic fat (Table 2)

There was a significant correlation between pancreatic endocrine impairment and the amount of pancreatic fat in the univariate analysis (OR = 0.61, 95% CI = 0.43–0.83, *P* = 0.0013). In the multivariate analysis, we included some metabolic factors or image findings that could be associated with pancreatic endocrine impairment as covariates; these were sex, age, body mass index, visceral fat area, hypertension or dyslipidemia as comorbidities, and presence of pancreatic calcifications. As it was presumed that diabetes as a comorbidity has a strong influence on the result of glucagon stimulation test, it was not included in the covariate. However, we decided to divide the participants based on the presence or absence of diabetes and further analyzed the target populations separately.

**Table 2:** Association between pancreatic endocrine impairment and the amount of pancreatic fat.

	OR (95% CI) *	*P* value
**All participants (n = 109**)		
**Univariate analysis**		
P/S ratio	0.61 (0.43–0.83)	0.0013
Multivariate analysis**		
P/S ratio	0.38 (0.22–0.61)	< .0001
**Participants without diabetes (n = 93)**		
**Univariate analysis**		
P/S ratio	0.70 (0.49–0.99)	0.04
Multivariate analysis**		
P/S ratio	0.39 (0.21–0.66)	0.0002
**Participants with diabetes (n = 16)**		
**Univariate analysis**		
P/S ratio	0.25 (0.03–0.85)	0.02

P/S ratio: mean CT value of pancreas/CT value of spleen, OR: odds ratio, CI: confidence interval.

*Odds ratio is shown per 0.1 unit increase

**The covariates were sex, age, body mass index, visceral fat area, hypertension or dyslipidaemia as comorbidities, and presence of pancreatic calcifications.

There was a significant correlation between pancreatic endocrine impairment and the amount of pancreatic fat in the multivariate analysis (OR = 0.38, 95% CI = 0.22–0.61, *P* < .0001) for all participants (n = 109).

Furthermore, similar statistically significant relationships were observed in both univariate (OR = 0.70, 95% CI = 0.49–0.99, *P* = 0.04) and multivariate (OR = 0.39, 95% CI = 0.21–0.66, *P* = 0.0002) analyses of the participants without diabetes (n = 93).

The relationship was also observed in univariate analysis (OR = 0.39, 95% CI = 0.21–0.66, *P* = 0.0002) for those with diabetes (n = 16), although multivariate analysis was not possible because of the number of participants.

### Association between pancreatic exocrine impairment and the amount of pancreatic fat

The amount of pancreatic fat was not associated with exocrine impairment in univariate analysis (OR = 0.80, 95% CI = 0.59–1.06, *P* = 0.12).

## Discussion

The results of this study indicate that fatty pancreas is positively associated with pancreatic endocrine impairment but not with pancreatic exocrine impairment.

**Fig 1:**
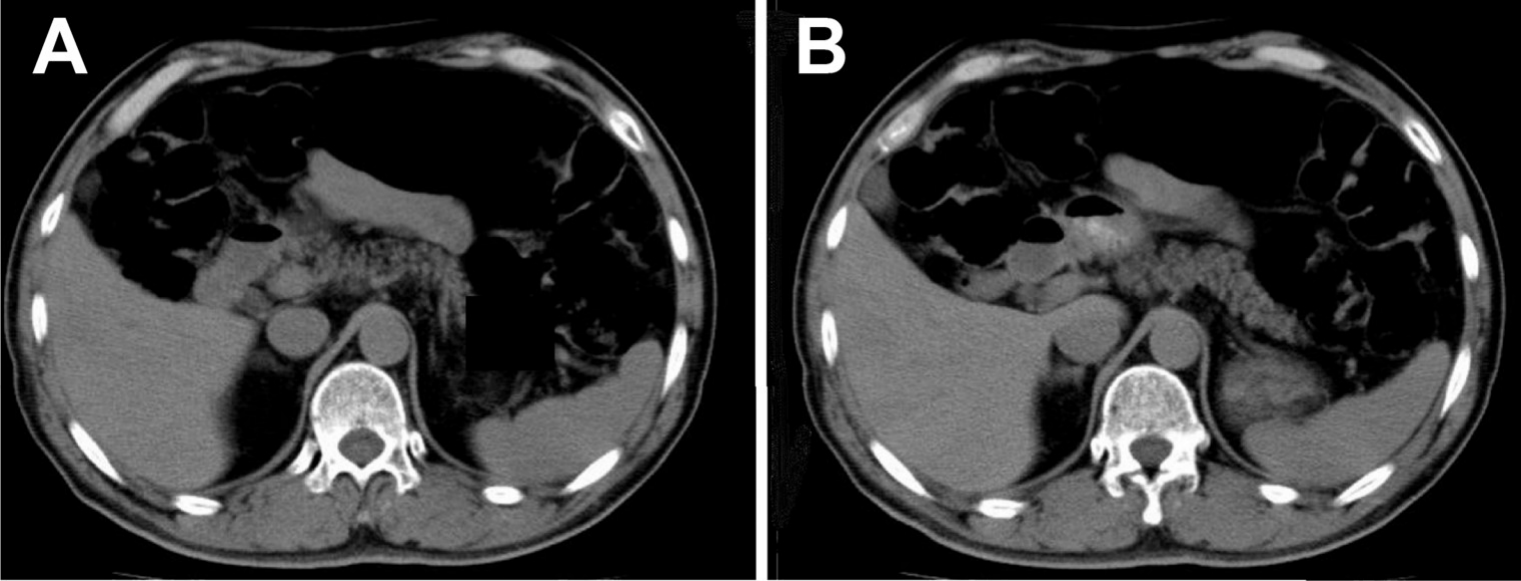
**CT Images of the pancreas in a 63-year**-**old man (A) (B).** The patient was relatively lean (body mass index = 22.5 kg/m^2^, visceral fat area = 61.6 cm^2^). The pancreas showed changes in fatty composition (mean CT value of pancreas = 24.4 Hounsfield units, mean CT value of pancreas / CT value of spleen = 0.53). The participant had pancreatic endocrine impairment (increment of C-peptide immunoreactivity level at 6 min after an intravenous injection of 1 mg glucagon = 1.39 mmol/L) and pancreatic exocrine impairment (urinary p-amino benzoic acid excretion rate = 62.5%).

The association between pancreatic fat and pancreatic endocrine impairment may be attributed to β-cell lipotoxicity [5,19]. However, as one review reported that fatty pancreas and type 2 diabetes are consequences of obesity [20], it is widely believed that fatty pancreas has a strong association with obesity. By contrast, the majority of the participants in this study were not obese but relatively lean (BMI [mean]: 22.0 kg/m2, visceral fat area [mean]: 71.1 cm2) as shown in Fig 1, and the result in this study indicates that fatty pancreas is an independent risk factor for pancreatic endocrine impairment, which is extremely important. Because there were few populations with diabetes in this study, further analysis of the influence of fatty pancreas on insulin secretion in diabetic patients is necessary.

It is necessary to further study the clinical course of the patients with pancreatic endocrine impairment due to fatty pancreas, which include whether the pancreatic endocrine impairment develop to diabetes.

Electron microscopy revealed fat droplets in acinar cells from rodents [21], and fatty pancreas could theoretically be associated with pancreatic exocrine impairment [1]. However, this association was not verified in this study. One reason could be attributed to the instability of the BT-PABA test. Studies comparing the results of the BT-PABA test with those of the secretin test showed that the sensitivity of the BT-PABA test was 71% in patients with advanced chronic pancreatitis and severe pancreatic exocrine impairment but only 46% in those with mild to moderate pancreatic exocrine impairment [22]. Further studies to investigate the association between fatty pancreas and pancreatic exocrine impairment with another method, such as measurement of fecal elastase 1, breath tests with ^13^C-labeled compounds [23] or a direct intubation test, are needed.

Fatty pancreas has received considerable attention in recent years, and research on this topic is increasing. Many risk factors for fatty pancreas have been reported. Fatty pancreas has a strong association with obesity [24–27] and aging [8,28]. It has also observed in some congenital diseases, such as cystic fibrosis [29–31] and Shwachman–Diamond syndrome [32], both of which are associated with deteriorating pancreatic exocrine function with aging.

Although a number of other risk factors for fatty pancreas have been reported in studies based on case reports or animal studies [19], any factors that can cause acinar cell necrosis may lead to fatty pancreas [1].

Our study had some limitations. Although single-voxel magnetic resonance spectroscopy (MRS) is considered the standard modality to assess fatty pancreas [19], we selected unenhanced CT for the assessment of pancreatic fat content in this study because of its ease of use in practical medicine. However, because fat content is heterogeneous in the pancreas [33], the measured ROIs of the pancreas may not accurately represent the fat content in the entire pancreas because of sampling error. Moreover, most patients who undergo the glucagon stimulation test or BT-PABA test are generally suspected of having any pancreatic diseases based on symptoms or imaging findings. Although we excluded patients with pancreatic neoplasm or autoimmune pancreatitis, it is possible that the characteristics of the participants in this study were somewhat different from those of the general population due to selection bias. Additional studies among general populations who do not have any symptoms or image findings of pancreatic diseases may be necessary.

In conclusion, fatty pancreas was found to be an independent risk factor for pancreatic endocrine impairment in this study. Not all of the patients who exhibited pancreatic endocrine impairment based on the glucagon stimulation test will develop diabetes. However, we must keep in mind that fatty pancreas is at least a risk factor for impaired insulin secretion and might be a clinical sign of diabetes. Based on the results of this study, we hope that patients with pancreatic endocrine impairment will be identified earlier in the future.

## Supporting information

S1 Database Data supporting findings in this study. (XLSX)
