# Correction: Association of fatty pancreas with pancreatic endocrine and exocrine function

**DOI:** 10.1371/journal.pone.0236915

**Published:** 2020-07-23

**Authors:** Hayato Miyake, Junichi Sakagami, Hiroaki Yasuda, Yoshio Sogame, Ryusuke Kato, Kanetoshi Suwa, Katsuyuki Dainaka, Tomoki Takata, Isao Yokota, Yoshito Itoh

The units of C peptide immunoreactivity (CPR) and Δ C peptide immunoreactivity (ΔCPR) appear incorrectly throughout the article. The correct units are [ng/ml].
